# Associations between variants of the *HAL* gene and milk production traits in Chinese Holstein cows

**DOI:** 10.1186/s12863-014-0125-4

**Published:** 2014-11-25

**Authors:** Haifei Wang, Li Jiang, Wenwen Wang, Shengli Zhang, Zongjun Yin, Qin Zhang, Jian-Feng Liu

**Affiliations:** Key Laboratory of Animal Genetics, Breeding and Reproduction, Ministry of Agriculture, National Engineering laboratory for Animal Breeding, College of Animal Science and Technology, China Agricultural University, Beijing, 100193 China; College of Animal Science and Technology, Anhui Agricultural University, Hefei, 230036 China

**Keywords:** *HAL* gene, Single nucleotide polymorphism, Milk production traits, Chinese Holstein cows

## Abstract

**Background:**

The histidine ammonia-lyse gene (*HAL*) encodes the histidine ammonia-lyase, which catalyzes the first reaction of histidine catabolism. In our previous genome-wide association study in Chinese Holstein cows to identify genetic variants affecting milk production traits, a SNP (rs41647754) located 357 bp upstream of *HAL*, was found to be significantly associated with milk yield and milk protein yield. In addition, the *HAL* gene resides within the reported QTLs for milk production traits. The aims of this study were to identify genetic variants in *HAL* and to test the association between these variants and milk production traits.

**Results:**

Fifteen SNPs were identified within the regions under study of the *HAL* gene, including three coding mutations, seven intronic mutations, one promoter region mutation, and four 3′UTR mutations. Nine of these identified SNPs were chosen for subsequent genotyping and association analyses. Our results showed that five SNP markers (ss974768522, ss974768525, ss974768531, ss974768533 and ss974768534) were significantly associated with one or more milk production traits. Haplotype analysis showed that two haplotype blocks were significantly associated with milk yield and milk protein yield, providing additional support for the association between *HAL* variants and milk production traits in dairy cows (*P* < 0.05).

**Conclusion:**

Our study shows evidence of significant associations between SNPs within the *HAL* gene and milk production traits in Chinese Holstein cows, indicating the potential role of HAL variants in these traits. These identified SNPs may serve as genetic markers used in genomic selection schemes to accelerate the genetic gains of milk production traits in dairy cattle.

**Electronic supplementary material:**

The online version of this article (doi:10.1186/s12863-014-0125-4) contains supplementary material, which is available to authorized users.

## Background

The dairy industry plays a key role in contributing to agricultural economy and it is the main financial source of dairy farmers. Hence an improvement in milk production traits continues to be the most profitable breeding goal. Although classical selection approaches have obtained great genetic gains of farm animal performance, the implementation of selection programs containing molecular information could facilitate the improvement in selection response of animals [1].

With the progress in DNA-based marker technology, the identification of genomic regions (i.e. quantitative trait loci, QTLs) that associated with complex traits has been available [2]. Incorporating the detected QTLs into genetic evaluation makes it possible to evidently improve the accuracy of selection, thus accelerating the genetic improvement of animal productivity. Over the last decades, numerous investigations regarding identification of QTLs for milk production traits in dairy cattle have been reported [3-5]. Advances in detecting causal genes for complex traits are slow as the linkage mapping results in large confidence intervals [6,7]. Particularly, the region that a QTL is mapped might contain too many plausible candidate genes. Until recently, only few candidate genes responsible for variation in complex traits were discovered and functionally confirmed in dairy cattle, i.e., the diacylglycerol acyltransferase 1 (*DGAT1*) gene [8] and the growth hormone receptor (*GHR*) gene [9].

High throughput SNP genotyping technology has made it feasible to screen for mutations associated with complex traits by genome-wide association study (GWAS). The advantages of GWAS are an increase in the power to detect underlying variants in complex traits and in simplifying the discovery of causal variants [10]. The GWAS has been widely recognized as a valid approach for gene discovery and successfully identified many genes associated with economically important traits in several domestic animal species [11-13].

In our previous GWAS study in Chinese Holstein dairy cows to identify genetic variants affecting milk production traits, we not only identified some previously reported functional genes but also discovered 105 SNPs that significantly associated with milk production traits [11]. Among these SNPs, a SNP, BFGL-NGS-110018 (*P* = 1.50E-07, n = 1815), located 357 bp upstream of the histidine ammonia-lyse (*HAL*) gene was highly significant in our GWAS results with effects on milk yield and milk protein yield. The *HAL* gene located on bovine chromosome (BTA) 5 encodes a cytosolic enzyme known as histidase or histidinase, which catalyzes the first reaction of histidine catabolism, the nonoxidative deamination of histidine to urocanic acid [14]. Histidine is the first-limiting amino acid for milk protein synthesis in dairy cows, the infusion of which was related to significant increases in milk and milk protein yields [15]. In addition, the *HAL* gene is located within the reported QTLs for milk production traits [16-18]. These findings strongly suggest that *HAL* is a promising positional and functional candidate gene for milk production traits in dairy cattle.

In this study, we explored genetic variants of the *HAL* gene and tested the association between *HAL* variants and milk production traits in Chinese Holstein cows, and performed functional prediction analysis of the identified variants. Significant associations were observed between a subset of *HAL* variants and the milk production traits of interest. Our study firstly identified the genetic variants within the *HAL* gene in dairy cattle and the information of these SNPs markers will be available for genomic selection in dairy cattle breeding programs.

## Methods

### Ethics statement

The whole study protocols for collection of the tissue samples of experimental individuals were reviewed and approved by the Institutional Animal Care and Use Committee (IACUC) of China Agricultural University.

### Animals and phenotypic data

Thirteen unrelated sires and their corresponding 638 daughters with known milk production phenotypes were collected to construct the study population. Family size ranges from 5 to 136 daughters with an average of about 49 daughters per sire. Daughters were from 15 Chinese Holstein cattle farms in Beijing, China, where regular and standard performance testing (Dairy Herd Improvement, DHI) has been conducted since 1999. The official up to date estimated breeding values (EBVs) of five milk production traits, including first lactation 305-d milk yield (MY), first lactation 305-d fat yield (FY), first lactation 305-d protein yield (PY), first lactation average 305-d fat percentage (FP), and first lactation average 305-d protein percentage (PP) were considered as phenotypic observations used for the subsequent analysis. These EBVs were calculated by the official Dairy Data Center of China based on the genetic parameters that estimated via the complete DHI data of Chinese Holstein cattle population.

### Genomic DNA extraction

Genomic DNA was isolated from blood sample of the daughters by QIAamp DNA blood kit (Qiagen, Hilden, Germany) according to the manufacturer’s instructions and from semen sample of the sires using the routine salt-out procedure. The quality and quantity of extracted DNA were measured using the spectrophotometer and gel electrophoresis.

### SNP identification and genotyping

A DNA pool was constructed by mixing equal amounts of DNA from the 13 unrelated sires to identify variants of the *HAL* gene. All exons and their adjacent intronic sequences were targeted for selective amplification by PCR. Sixteen pairs of nucleotide primers (Additional file 1: Table S1) targeting the regions of interest were designed using Primer Premier 5.0 [19] based on the reference sequence NC_007303.5 and synthesized by Sangon Biotech Co. Ltd. (Shanghai, China).

PCR amplifications were performed in a total volume of 25 μL. The following reagents were used for amplification: 50–100 ng of genomic DNA, 10 pmol of each primer, 5 mM of dNTP mix, 2.5 μL of 10× PCR buffer, 0.625 U Taq DNA Polymerase (Takara Biotechnology (Dalian) Co., Ltd., Dalian, China). The PCR reaction conditions were as follows: 95°C for 5 min, followed by 35 cycles of 94°C for 30 sec, annealing from 46°C to 58°C for 30 sec, 72°C for 35 sec, and a final extension at 72°C for 10 min. The PCR products were directly sequenced using an ABI3730xl DNA analyzer (Applied Biosystems, Foster City, CA, USA).

We chose nine out of these identified SNPs focusing primarily on SNPs located in the coding regions for subsequent genotyping in 638 Chinese Holstein cows using the iPLEX MassARRAY system (Sequenom Inc., San Diego, California, USA).

### Statistical analyses

To study the effects of *HAL* variants on milk production traits, we performed a single locus-based regression analysis. To this end, we utilized the same analytical method as employed in our previous GWAS study [11]. A linear mixed regression model was adapted as follows:

1 $$ \mathbf{y}=\mathbf{1}\mu +b\mathbf{x}+\mathbf{Z}\mathbf{a}+\mathbf{e} $$

where **y** is the vector of EBVs of all daughters, *μ* is the overall mean, b is the regression coefficient of EBVs on SNP genotypes. **x** is the vector of the SNP genotype predictors set as 0, 1 or 2, representing the three genotypes 11, 12 and 22 respectively (setting 1 as the minor frequency allele). **a** is the vector of residual polygenetic effects with $\mathrm{a}\sim \mathrm{N}\left(0,\mathbf{A}{\delta}_a^2\right)$ (where **A** is the additive genetic relationship matrix according to the pedigree data regarding all experimental individuals and ${\delta}_a^2$ is the additive variance), **Z** is the incidence matrix of **a**, and **e** is the vector of residual errors with $\mathrm{e}\sim \mathrm{N}\left(\mathbf{0},\mathbf{W}{\updelta}_{\mathrm{e}}^2\right)$ (where W is a diagonal matrix with the diagonal elements equal to 1/REL_ij_, of which REL_ij_ is the reliability of EBV of daughter i in family j, and ${\delta}_{\mathrm{e}}^2$ is the residual error variance). For each SNP, the estimate of *b* and the corresponding sampling variance $\mathrm{V}\mathrm{a}\mathrm{r}\left(\widehat{\mathrm{b}}\right)$ were obtained by solving the mixed model equations. Afterwards, a Wald chi-squared statistic ${\widehat{\mathrm{b}}}^2/\mathrm{V}\mathrm{a}\mathrm{r}\left(\widehat{\mathrm{b}}\right)$ with df = 1 was applied to determine whether the SNPs were associated with the milk production traits considered in this study.

Pairwise linkage disequilibrium (LD) was calculated and plotted by using Haploview [20]. Haplotype block structure was defined for marker pairs showing D’ > 0.75, with the fraction of strong linkage disequilibrium informative comparisons set at 0.9. For haplotype-based association analysis, considering multiple loci in high LD, we extended the original haplotype trend regression (HTR) approach [21] by allowing random effects in the regression model. The haplotype trend regression model with polygenic random effects, as described in our previous work [22], was as following:

2 $$ \mathbf{y}=\mathbf{1}\mu +\mathbf{X}\mathbf{h}+\mathbf{Z}\mathbf{a}+\mathbf{e} $$

where **y** is the vector of EBV of all experimental daughters, *μ* is the overall mean. **1** is the n-dimensional vector with all elements equal to 1, h is the haplotype fixed effect vector with elements h_i_ (i = 1, 2, …, k) being the effect of haplotype of all k distinct haplotypes within the haplotype block. **X** is the n × k indicator matrix with the same pattern as that in [21], **a** is the vector of the residual polygenetic effects with $\mathrm{a}\sim \mathrm{N}\left(0,\mathbf{A}{\delta}_a^2\right)$ (where **A** is the additive genetic relationship matrix based on the pedigree data regarding all experimental individuals and ${\delta}_{\mathrm{a}}^2$ is the additive variance), **Z** is the incidence matrix of **a**, and **e** is the vector of residual errors with $\mathrm{e}\sim \mathrm{N}\left(\mathbf{0},\mathbf{W}{\updelta}_{\mathrm{e}}^2\right)$ (where ${\delta}_{\mathrm{e}}^2$ is the residual error variance and the weight matrix **W** is a diagonal matrix with each diagonal element equal to the reciprocal of the reliability of the EBV of individual i). With regard to each haplotype block, the estimate of the haplotype effects vector h and the corresponding sampling variance Var(ĥ ' h) were acquired via solving the mixed model equations, and a Wald chi-squared statistic $\frac{\widehat{\mathrm{h}}\hbox{'}\mathrm{h}}{\mathrm{Var}\left(\widehat{\mathrm{h}}\hbox{'}\mathrm{h}\right)}$ with df = k-1 (k is the number of distinct haplotypes in the experimental populations within the region of haplotype block investigated) was constructed to examine whether the haplotype block was associated with the trait.

In the haplotype analysis, the haplotype with frequency > 5% was regarded as a distinguishable haplotype, and those haplotypes each with relative frequency < 5% were pooled into a single group. For both analyses, false discovery rate (FDR) correction was performed to correct for multiple testing. An adjusted P-value of ≤ 0.05 was considered statistically significant.

Hardy-Weinberg equilibrium (HWE) test was performed on each SNP. We calculated the SNP allele frequencies by determining their proportions in the samples and then estimated the expected genotype numbers using the expected genotype frequencies under HWE. Then, a goodness-of-fit test (Chi-square) was used to compare the expected and observed genotype numbers, choosing a significance level of 0.05.

### Quantification of gene expression

Total RNA was extracted from the mammary gland tissues using TRIzol Reagent (Life technology, Carlsbad, CA, USA) following the manufacturer’s protocols. The tissue samples were collected from eight genetically unrelated Chinese Holstein cows in late lactation (approximately 300 days) after slaughter within 30 min and frozen in liquid nitrogen and then stored at −80°C. The quality of extracted RNA was assessed by electrophoresis in 1% agarose gel and quantified with the NanoDropTM 2000 spectrophotometer (Thermo Scientific, Wilmington, DE, USA). RNA was then purified and reversely transcribed into cDNA using PrimerScript® RT reagent Kit with gDNA Eraser (Takara Biotechnology (Dalian) Co., Ltd.) following the manufacturer’s instructions. Quantities of mRNA were then measured with real-time quantitative PCR (qPCR) using a LightCycler® 480 Real-Time PCR System (Roche, Hercules, CA, USA). The qPCR assays were performed with a volume of 20 μL containing 10 μL SYBR Green Mixture, 7 μL deionized water, 1 μL template of cDNA, 1 μL of each primer. The reaction conditions were as follows: 95°C for 5 min, 45 cycles of 95°C for 10 sec, 60°C for 10 sec, 72°C for 10 sec. The glyceraldehyde-3-phosphate dehydrogenase gene (*GAPDH*) was used as a reference to normalize gene expression. Primers for the amplification of both *HAL* and *GAPDH* genes are presented in Additional file 2: Table S2. Each qPCR assay was carried out in triplicate. The relative gene expression was calculated by using the 2^‐ *ΔΔ*Ct^ method [23].

### Bioinformatics analysis of identified SNPs

The potential impact of the amino acid transition on the structure or function of the protein was predicted using the two web server tools SIFT (http://sift.jcvi.org/www/SIFT.html) and PolyPhen (http://genetics.bwh.harvard.edu/pph2/). The search for potential transcription factor binding site (TFBS) for polymorphisms around promoter region was performed by using the online tool TFSEARCH (http://www.cbrc.jp/research/db/TFSEARCH). Effects of 3′UTR variants on microRNA binding sites were searched with STarMir [24].

## Results

### SNPs within the *HAL* gene in Chinese Holstein cows

A total of fifteen SNPs in the *HAL* gene were discovered in this study (Table 1). Among these identified SNPs, seven were found within introns and one within the promoter region. In addition, seven SNPs were exonic, of which two were synonymous substitutions and one was a non-synonymous switch resulting in an amino acid substitution (Gly to Glu) at residue 228, and the remaining four were located in the 3′UTR.

**Table 1:** **SNPs detected by sequencing in the** ***HAL*** **gene**

**NO.**	**Gene region**	**Position on Btau_4.6.1**	**Submission number**	**Nucleotide substitution**	**Amino acid substitution**
1	Promoter	64547553	ss974768522	C > T	
2	Exon1	64547394	ss974768523	T > C	N42N
3	Intron2	64546261	ss974768524	T > C	
4	Exon5	64545391	ss974768525	C > T	I156I
5	Intron5	64545371	ss974768526	G > T	
6	Exon8	64543949	ss974768527	G > A	G228E
7	Intron11	64537723	ss974768528	C > G	
8	Intron11	64537682	ss974768529	A > G	
9	Intron15	64534230	ss974768530	C > T	
10	Intron17	64529509	ss974768531	T > C	
11	Intron17	64529497	ss974768532	C > A	
12	3′UTR	64525640	ss974768533	C > A	
13	3′UTR	64524579	ss974768534	C > T	
14	3′UTR	64524558	ss974768535	G > A	
15	3′UTR	64524557	ss974768536	T > C	

**Figure 1:**
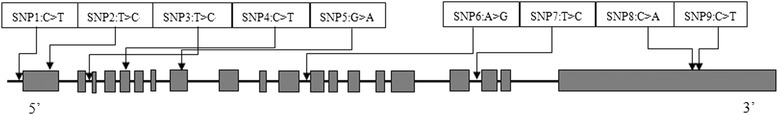
**Locations of the nine genotyped SNPs in the** ***HAL*** **gene.** The grey bars denote exons.

SNPs in coding parts of exons are considered to be more likely to be functional than those in other genic regions. We therefore chose nine SNPs mainly located in the coding regions for genotyping (Figure 1). Locations and allele frequencies of the nine SNPs are shown in Table 2.

**Table 2:** **Genotypes, allele frequencies and the significance of deviations from HWE** ^**1**^

**Submission number**	**Location of SNP**	**Position on Btau_4.6.1**	**Allele substitution**	**Genotype**	**Genotype frequencies**	**MAF** ^**2**^	***P*** **-value** ^**3**^
974768522	Promoter	64547553	C > T	CC	0.426	0.339	0.253
				CT	0.468		
				TT	0.106		
974768523	Exon1	64547394	T > C	TT	0.527	0.281	0.257
				TC	0.385		
				CC	0.086		
974768524	Intron2	64546261	T > C	TT	0.588	0.251	0.0004
				TC	0.323		
				CC	0.089		
974768525	Exon5	64545391	C > T	CC	0.647	0.196	0.898
				CT	0.314		
				TT	0.039		
974768527	Exon8	64543949	G > A	GG	0.523	0.282	0.307
				GA	0.389		
				AA	0.088		
974768529	Intron11	64537682	A > G	AA	0.078	0.267	0.370
				AG	0.378		
				GG	0.544		
974768531	Intron17	64529509	T > C	TT	0.433	0.327	0.018
				TC	0.481		
				CC	0.086		
974768533	3′UTR	64525640	C > A	CC	0.091	0.307	0.680
				CA	0.433		
				AA	0.476		
974768534	3′UTR	64524579	C > T	CC	0.486	0.304	0.850
				CT	0.420		
				TT	0.094		

^1^HWE = Hardy-Weinberg equilibrium.

^2^MAF = Minor allele frequency.

^*3*^P-value was computed for each SNP by chi-square test.

Compared with the SNP loci contained in the 700 K BovineHD SNP array, none of these identified SNPs are currently present. The details of all identified SNPs have been submitted to dbSNP (http://www.ncbi.nlm.nih.gov/SNP/) and will be publicly available (submission numbers from ss974768522 to ss974768536) in dbSNP Build (B142).

### Association analyses

Our findings showed that significant associations exist between five markers (ss974768522, ss974768525, ss974768531, ss974768533 and ss974768534) and one or more milk production traits (FDR *P* < 0.05). Out of these associations, the synonymous SNP ss974768525 was associated with FY, with the genotype *CC* showing higher levels in FY. Two 3′UTR variants, ss974768533 and ss974768534, showed significant associations with PP with the genotype *CC* linked to higher increases in PP. The promoter variant ss974768522 was significantly associated with PY with the heterozygous genotype CT related to higher increases in this trait. The intronic mutation ss974768531 was also found to be associated with MY, FY, PY and FP. A marginal significant association between SNP ss974768527 and PY was observed (P = 0.0447, not significant after correction for multiple testing). Results of SNP marker associations with milk production traits are presented in Additional file 3: Table S3.

**Figure 2:**
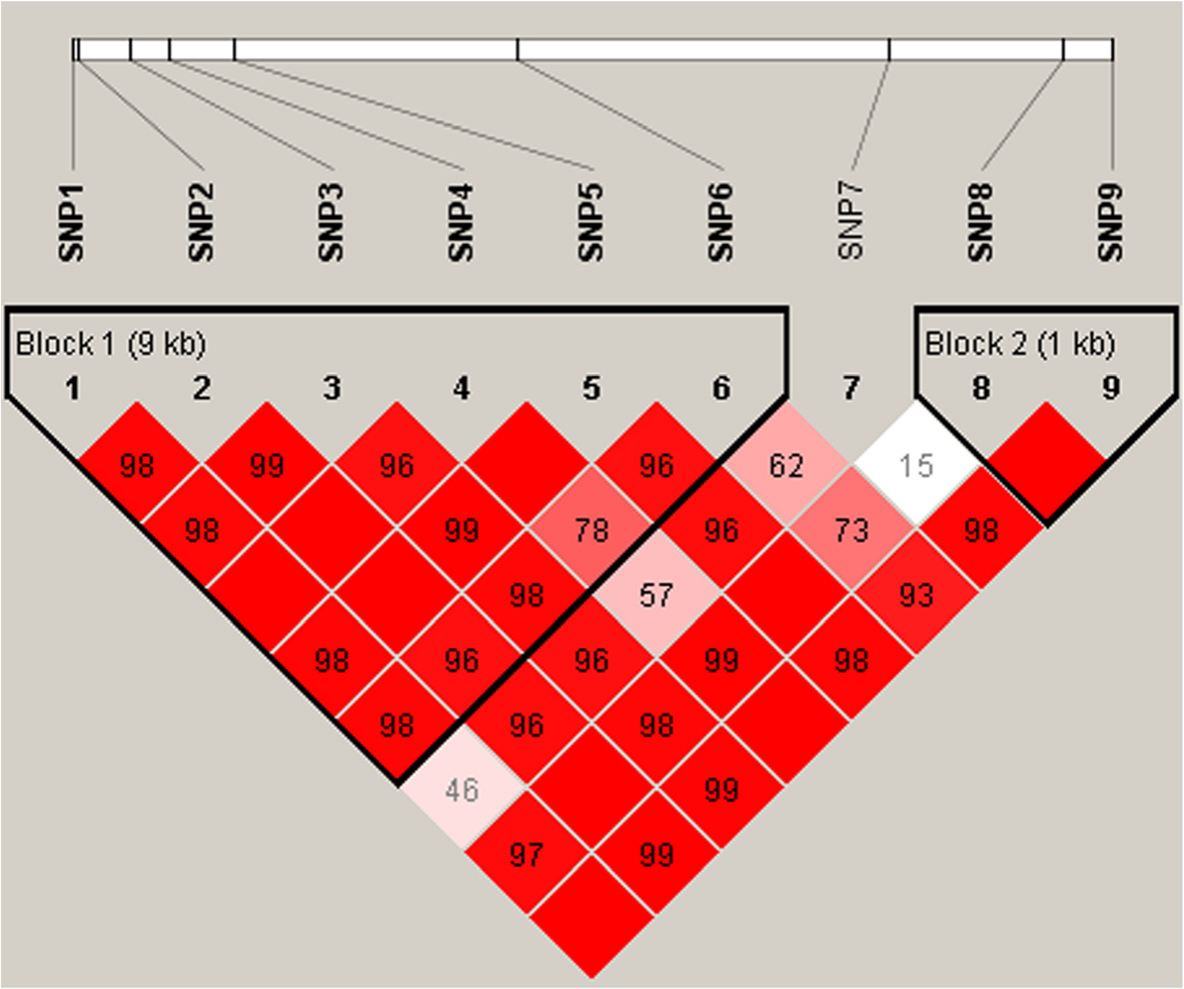
**Linkage disequilibrium (LD) pattern for the nine genotyped SNPs within the HAL gene.** LD blocks are denoted with triangles. The values in diamond are D’ × 100 between pairs of SNPs. Empty diamonds indicate D’ = 1 (i.e. complete LD between two SNPs).

Two haplotype blocks were constructed (Figure 2). The block 1 included 6 SNPs, which formed 5 major haplotypes (> 5%) in our resource population. The block 2 consisted of 3 major haplotypes. The constitution of the haplotypes and their frequencies are listed in Table 3. The haplotype blocks containing these SNPs were also tested for associations with milk production traits. Both the two blocks were associated with MY and PY (*P* < 0.05) (Table 3). These findings produced additional support for the association between these SNP markers and milk production traits.

**Table 3:** **Haplotypes, their frequencies and associations with milk production traits in Chinese Holstein cows**

**Haplotypes**	**SNP1 C > T**	**SNP2 T > C**	**SNP3 T > C**	**SNP4 C > T**	**SNP5 G > A**	**SNP6 A > G**	**SNP7 A > C**	**SNP8 C > T**	**Frequency (%)**	**MY**		**PY**	
										**P-value**	**APV**	**P-value**	**APV**
TTTCGG	T	T	T	C	G	G			33.9	2.38E-03	2.85E-03^*^	3.31E-03	6.26E-03^*^
CCCCAG	C	C	C	C	A	G			24.8				
CTTTGA	C	T	T	T	G	A			16.6				
CTTCGA	C	T	T	C	G	A			9.4				
CTTCGG	C	T	T	C	G	G			9.1				
Pooled haplotypes	C	T	T	T	G	G			3.0				
	C	C	T	C	A	G			2.4				
	C	C	T	C	A	A			0.07				
	C	T	T	C	A	G			0.01				
AC							A	C	38.8	6.75E-04	1.87E-03^*^	7.78E-03	9.03E-03^*^
CC							C	C	30.8				
AT							A	T	30.4				

SNP1 to SNP8 represent SNPs with the submission numbers of 974768522, 974768523, 974768524, 974768525, 974768527, 974768529, 974768533 and 974768534 respectively. APV = Adjusted P-value, *APV indicates the significant associations after false discovery rate correction for multiple testing.

### Expression analysis of the *HAL* gene

**Figure 3:**
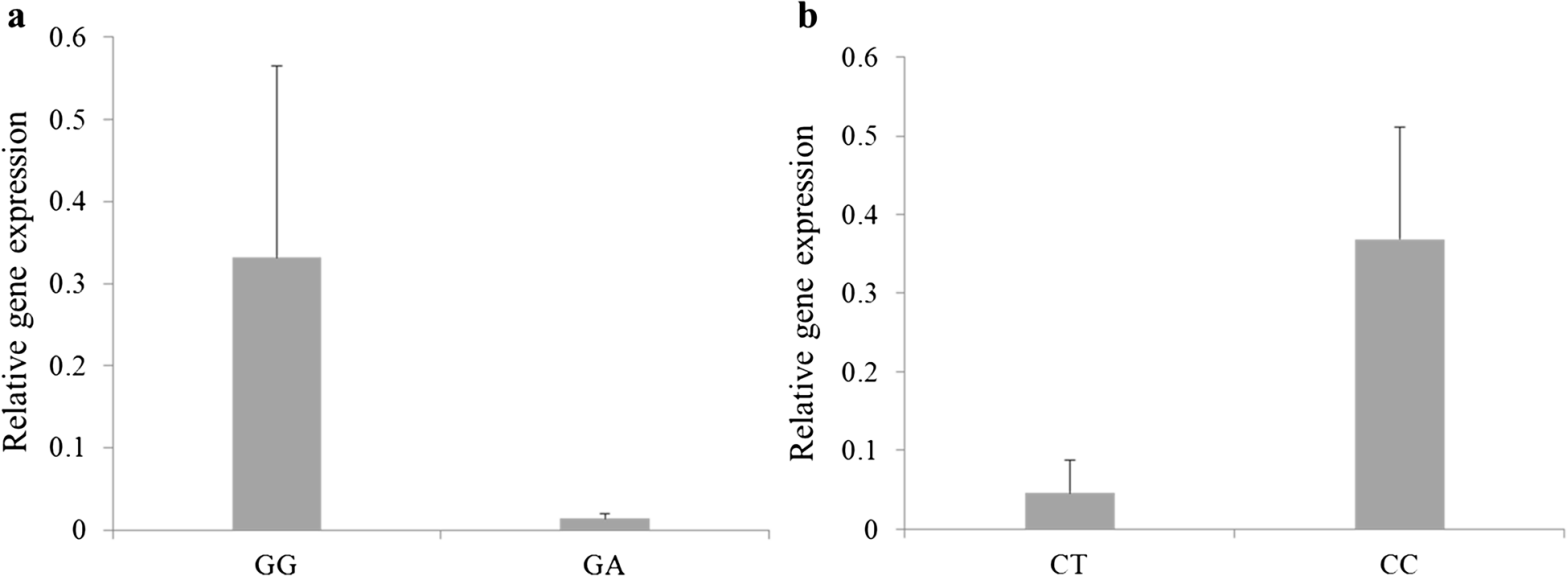
**Relative gene expression in the mammary glands with different genotypes at the loci of SNPs (a) ss974768522 and (b) ss974768527.** The internal reference gene *GAPDH* was used for normalization. Bars indicate mean ± SE (n = 4). SE = standard error.

As the possible effects of non-synonymous mutations on gene expression by altering mRNA stability [25], we measured *HAL* expression in mammary gland tissues with different genotypes at the locus of SNP ss974768527. Results indicated that individuals with the *GG* genotype have greater expression level (n = 4, mean relative expression = 0.331) than those with the *GA* genotype (n = 4, mean relative expression = 0.014) (Figure 3a). We also analyzed the possible effects of SNP ss974768522 located in the regulatory region on gene expression and observed that individuals with the *CC* genotype have higher expression level (n = 4, mean relative expression = 0.368) than those with the *CT* genotype (n = 4, mean relative expression = 0.046) (Figure 3b). As the SNP ss974768527 was found to be a non-significantly associated mutation, the possible effects of SNP ss974768522 may be responsible for the observed differential gene expression results. For both SNPs, as the relatively lower frequency of the homozygous mutant genotype, we failed to obtain all genotypes in the tested samples.

### Prediction of potentially functional SNPs

Effects of the detected non-synonymous mutation ss974768527 were predicted to be “tolerated” and “benign” by the SIFT and PolyPhen programs respectively, implying that the related protein structure and function may not be affected.

**Figure 4:**
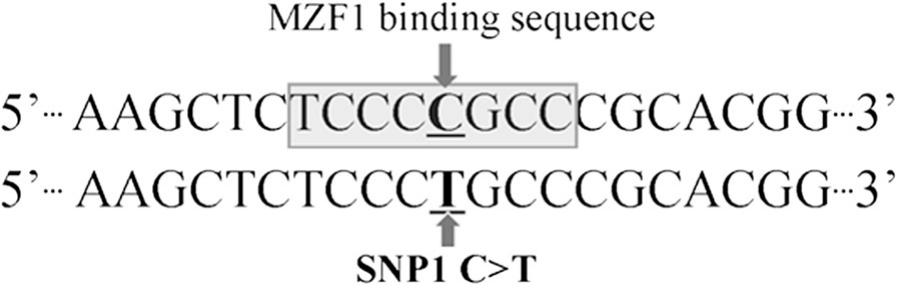
**The effects of allele substitution on the affinity to the** ***MZF1*** **binding motif.**

We also examined the impact of promoter variant ss974768522 on the binding site for transcriptional factors. Results showed that an 8 bp sequence containing the *C* allele was within the transcription factor myeloid zinc finger 1 (MZF1) binding motif, which was disrupted by the presence of the *T* allele (Figure 4).

In addition, the STarMir analysis showed that none of the four SNPs within 3′UTR is situated within the binding site for mature microRNAs in cattle.

## Discussion

In this study, based on the reported QTLs for milk production traits [16-18] and our previous GWAS findings [10], we explored the genetic variants of the *HAL* gene and tested their association with milk production traits in Chinese Holstein cows. Our results demonstrated that significant associations exist between *HAL* variants and milk production traits, which provide important SNP marker information that can be considered for genetic improvement in dairy breeding schemes.

The identified non-synonymous SNP ss974768527 caused an amino acid substitution (Gly to Glu) at residue 228 of the protein. These two amino acids are both polar amino acids having aliphatic side chain groups. This substitution does not seem to influence the structure and function of the related protein, which is further supported by the prediction of functional SNPs. Therefore this non-functional change may explain in part why no significant association was observed between this SNP and milk traits under study.

As synonymous mutations do not change the amino acid sequence of the encoded proteins, this type of genetic variation has generally been considered as silent mutation with no effect on protein structure and function. However, accumulating evidence indicates that synonymous SNPs could have functional consequences [26]. Considerable studies have identified the significant contribution of synonymous SNPs to human disease [27-29]. Synonymous SNPs could influence protein expression and enzymatic activity through modifying mRNA stability [30,31]. In addition, synonymous codons may have different codon usage, and therefore lead to varied gene expression [32]. According to the codon usage table of cattle (http://www.kazusa.or.jp/codon/), the two synonymous SNPs detected here resulted in moderate alterations in codon usage frequency, for SNP (ss974768523) with AAT changed to AAC from 14.7 to 21.4 per thousand, for SNP (ss974768525) with ATC changed to ATT from 23.3 to 14.6 per thousand. Therefore, the two synonymous mutations identified in *HAL* might have the potential to influence gene expression and thus function of gene product.

One of the most important mechanisms by which SNPs in the regulatory region of a gene can affect transcription rate is by abolishing or introducing a TFBS [33]. In this study, the C/T substitution in the promoter region (ss974768522) of *HAL* may lead to the disappearance of binding sites of transcription factor MZF1, which pertains to the kruppel family of zinc-finger proteins. It has been identified as a bi-functional transcription regulator, repressing transcription in non-hematopoietic cells and activating transcription in hematopoietic cells [34]. Therefore, the possible effects on MZF1 may be responsible for the observed differential gene expression results.

In the present study, the EBVs of daughters were treated as phenotypic observations for association analyses. In addition to EBVs, de-regressed EBVs and yield deviation of individual were also often considered as phenotypes in the association study relevant to milk production traits [35,36]. Considering the effects of such phenotypes on QTL mapping and marker-assisted selection, none of them has a remarkably greater advantage over the others [37]. Consequently, we only used the EBVs for association analysis. It is also notable that we fulfilled the association analyses with the method of SNP by SNP individually by regressing the phenotypes of a single trait on either the genotypes of a SNP. As the multi-marker analysis containing multiple marker information was generally thought more powerful than single marker analysis [38], we further performed the haplotype-based association analysis to investigate the association of *HAL* variants with milk production traits.

SNP-based association analysis revealed that five out of the nine tested SNPs were associated with the milk production traits under analysis. Haplotype-based association analysis provided further evidence for these associations. As these identified SNPs are currently absent in the 700 K BovineHD SNP array, our findings will be helpful for the enrichment of genetic marker information used in genomic selection program of dairy cattle. The information of these identified SNPs could be integrated into the panel of high density SNP arrays used in genomic selection of dairy cattle breeding schemes to improve the frequency of the genetic marker which is positively correlated with the milk production traits of interest.

## Conclusion

In summary, we firstly explored genetic variants within the *HAL* gene and uncovered associations between the tested SNPs and milk production traits in Chinese Holstein cows. Results of this study could be used as a first step to explore *HAL* variants potentially responsible for variation in milk production traits and further studies will be required to replicate these association findings in different cattle populations. The information of these variants will be available for genomic selection to accelerate the genetic gains in Chinese Holstein cattle breeding programs.

## Additional files

Additional file 1: Table S1. Primers used for SNP identification in the *HAL* gene. Additional file 2: Table S2. Primers used for the amplification of bovine HAL and GAPDH genes by qPCR. Additional file 3: Table S3. Associations of SNP marker with estimated breeding values (EBVs) of milk production traits in dairy cows.
