# Supplementary material for: Associations between variants of the HAL gene and milk production traits in Chinese Holstein cows
Source: BMC Genet. 2014 Nov 25;15:125. doi: 10.1186/s12863-014-0125-4 (PMC4253992; doi:10.1186/s12863-014-0125-4)
Supplement: Additional file 1: Table S1. — Primers used for SNP identification in the HAL gene. [file 12863_2014_125_MOESM1_ESM.docx]

| Amplified region | Primer | Primer sequence (5’-3’) | Product size (bp) | Annealing (°C) |
| --- | --- | --- | --- | --- |
| Exon1 | 1F  1R | GGCAAAACTCAAATGGACAC TACACAGGAAGGACAGGGAC | 506 | 55.0 |
| Exon2&3  Exon4&5&6 | 2F  2R  3F | GCTGTCTGAAAGCATCTG  CCCTGCTCACTGCAACTA  TTGATTCTCCTCGATGCT | 585  635 | 51.3  50.6 |
| Exon7  Exon8  Exon9  Exon10  Exon11  Exon12&13  Exon14  Exon15&16  Exon17  Exon18&19  Exon20  Exon20  Exon20 | 3R  4F  4R  5F  5R  6F  6R  7F  7R  8F  8R  9F  9R  10F  10R  11F  11R  12F  12R  13F  13R  14F  14R  15F  15R  16F  16R | AGCCTCACCCTCCTTGTC  TACAGACAAGGAGGGTGA  GAGATACTGAGGGCTGAC  GGCAACTACCTGAACCAA  CACCACCCTGTCAATCAA  GGTGTCTATGCCATTTGT  GAGTGGGAATGCTTTGTC  CCTAGAACCCAGTATTCA  GTGAGGCTGATATTGAGA  TAAACAAGCATCACTGCCCTG ATTTTCTTCTTCCCCACCATC  TCAGGACCAGGTCAGCAC  TTCAAAGGGAAACAAGCA  TGACAGGCACCAGACTCA  GAGGGTTGCCACAATCAC  TTCTCCGACATGCTTACT  ACCTCCCTATTTACAACCT  TGGAACATCTAGTTCTTTGA  ATAGGGTTTGTTAAGTCCA  CTCGGCTTCTCAGCATTA  AACCCAGGGACTGAACAT  CCCATACTGAATCCTCTA  TTACTCTTCTCACCCTTA  ACAGTGTTTCCAGGTTAT  GAGGTCCTTTATCTTCTTC  TGGGCCTCAGGATGTTAC  ATGATGCCTCCAGCTCTT | 287  307  371  389  411  557  510  998  452  600  718  750  837 | 50.0  51.7  51.0  46.5  58.0  50.1  50.9  50.5  51.5  53.2  46.0  46.2  51.7 |

Table S1: Primers used for SNP identification in the *HAL* gene.
