# Supplementary material for: Associations between variants of the HAL gene and milk production traits in Chinese Holstein cows
Source: BMC Genet. 2014 Nov 25;15:125. doi: 10.1186/s12863-014-0125-4 (PMC4253992; doi:10.1186/s12863-014-0125-4)
Supplement: Additional file 2: Table S2. — Primers used for the amplification of bovine HAL and GAPDH genes by qPCR. [file 12863_2014_125_MOESM2_ESM.docx]

| Gene symbol | GenBank accession | Primer sequence (5’-3’) | Size (bp) | Annealing (°C) |
| --- | --- | --- | --- | --- |
| HAL | NM_001105440 | Forward: TCAAGGGCACCACCAAAGC  Reverse: GGACCGAAACCGAAAAGCA | 89 | 60 |
| GAPDH | NM_001034034 | Forward: GGTGCTGAGTATGTGGTGGA  Reverse: GGCATTGCTGACAATCTTGA | 180 | 60 |

Table S2: Primers used for the amplification of bovine *HAL* and *GAPDH* genes by qPCR.
