# Supplementary material for: Associations between variants of the HAL gene and milk production traits in Chinese Holstein cows
Source: BMC Genet. 2014 Nov 25;15:125. doi: 10.1186/s12863-014-0125-4 (PMC4253992; doi:10.1186/s12863-014-0125-4)
Supplement: Additional file 3: Table S3. — Associations of SNP marker with estimated breeding values (EBVs) of milk production traits in dairy cows. [file 12863_2014_125_MOESM3_ESM.docx]

Table S3: Associations of SNP marker with estimated breeding values (EBVs) of milk production traits in dairy cows.

| SN^1^ | Genotype | | MY | FY | PY | FP | PP |
| --- | --- | --- | --- | --- | --- | --- | --- |
| 974768522  974768523  974768524  974768525  974768527  974768529  974768531  974768533  974768534 | | CC  CT  TT  P-value  APV^2^  TT  TC  CC  P-value  APV^2^  TT  TC  CC  P-value  APV^2^  TT  TC  CC  P-value  APV^2^  AA  AG  GG  P-value  APV^2^  AA  AG  GG  P-value  APV^2^  TT  TC  CC  P-value  APV^2^  CC  CA  AA  P-value  APV^2^  CC  CT  TT  P-value  APV^2^ | 360.68±150.70  493.85±150.21  469.6±161.26  0.0126  0.0567  445.35±149.64  433.50±151.51  368.85±164.69  0.6380  0.7177  435.19±149.36  453.79±152.31  435.19±149.36  0.6208  0.7982  152.21±183.21  453.48±151.64  447.60±149.64  0.0300  0.0900  367.29±164.69  426.41±151.49  449.60±149.65  0.5783  0.8674  241.75±166.02  443.91±150.81  463.02±150.18  0.0300  0.0675  305.36±150.68^A^  543.52±150.02^B^  434.88±164.88^AB^  9.33E-07  8.39E-06^*^  299.39±163.41  451.19±150.32  452.75±150.69  0.1351  0.2432  446.05± 149.90  433.14± 151.04  376.14± 163.78  0.6742  0.6742 | 1.60±4.77  4.97±4.75  8.06±5.14  0.0124  0.0558  5.02±4.73  2.63±4.80  0.35±5.26  0.1245  0.1867  4.65±4.72  3.01±4.82  4.65±4.72  0.2263  0.2546  -2.41±5.90  1.86±4.80  5.52±4.73  0.0160  0.0480^*^  0.33±5.26  2.59±4.80  5.05±4.73  0.1147  0.2065  0.26±5.31  2.80±4.77  5.41±4.75  0.0830  0.1868  0.89±4.77^A^  5.63±4.74^B^  9.07±5.27^B^  0.0008  0.0072^*^  1.67±5.21  4.06±4.75  4.22±4.77  0.6215  0.6215  4.97±4.74  2.98±4.78  0.62±5.23  0.1853  0.2382 | 9.18±4.32^A^  13.76±4.30^B^  12.15±4.61^AB^  0.0019  0.0085^*^  12.50±4.29 11.28±4.34  7.02±4.71  0.0537  0.0690  12.05±4.28  12.07±4.36  12.05±4.28  0.0761  0.0856  5.02±5.23  13.09±4.34  11.39±4.29  0.0396  0.0713  6.99±4.71  11.13± 4.34  12.59±4.29  0.0447  0.0670  6.16±4.75  12.90±4.32  11.64±4.30  0.0202  0.0606  8.53±4.32^A^  14.33±4.30^B^  11.35±4.72^AB^  3.64E-05  3.28E-04^*^  10.18±4.67  12.56±4.31  11.08±4.31  0.3848  0.3848  12.55±4.29  11.50±4.33  6.51±4.69  0.0257  0.0578 | -11.82±4.98  -13.16±4.96  -8.87±5.39  0.2464  0.5544  -11.23±4.94  -13.53±5.01  -13.49±5.52  0.3620  0.5430  -11.25±4.92  -13.93±5.04  -13.64±5.51  0.2698  0.4856  -8.01±6.23  -15.19±5.01  -10.76±4.93  0.0184  0.0828  -13.40±5.52  -13.30±5.00  -11.36±4.94  0.4725  0.6075  -8.85±5.57  -13.65±4.98  -11.51±4.96  0.2014  0.6042  -10.40±4.98^a^  -14.41±4.95^b^  -6.51±5.53^a^  0.0041  0.0369^*^  -9.24±5.47  -12.57±4.96  -12.46±4.98  0.4886  0.5497  -11.36±4.95  -13.08±4.99  -13.39±5.49  0.5367  0.5367 | -2.30±2.26  2.00±2.25  -2.77±2.43  0.7717  0.7717  -1.69±2.24  -2.57±2.27  -4.68±2.48  0.0454  0.0817  -1.82±2.24  -2.42±2.29  -4.74±2.48  0.0538  0.0692  0.15±2.77  -1.40±2.28  -2.85±2.24  0.0525  0.0787  -4.67±2.48  -2.48±2.27  -1.73±2.24  0.0562  0.0632  -1.57±2.50  -1.25±2.26  -3.11±2.25  0.0282  0.0634  -1.10±2.26  -3.07±2.25  -2.52±2.48  0.0173  0.0519  0.62±2.46^A^  -1.81±2.25^AB^  -3.35±2.26^B^  0.0025  0.0225^*^  -1.66±2.25^Aa^  -2.30±2.27^a^  -5.44±2.47^Bb^  0.0079  0.0355^*^ |

All EBVs data are presented as least square means±standard error. ^A,B^Within the same column with different superscripts means *P* < 0.01, ^a,b^within the same column with different superscripts means *P* < 0.05. ^1^SN = Submission number. ^2^APV = Adjusted P-value, ^*^APV indicates the significant associations after false discovery rate correction for multiple testing.
